# Potential Lead Risk and Water Consumption Behavior in the Chicago Area: A Coordinated Oral Health Promotion (CO-OP) Study Analysis

**DOI:** 10.3390/ijerph23020193

**Published:** 2026-01-31

**Authors:** Natalie Crnosija, Kathleen R. Diviak, Molly Martin

**Affiliations:** Institute for Health Research and Policy, School of Public Health, University of Illinois Chicago, Chicago, IL 60607, USA; kdiviak@uic.edu (K.R.D.); mollyma@uic.edu (M.M.)

**Keywords:** water choice, lead risk, risk perception

## Abstract

**Highlights:**

**Public health relevance—How does this work relate to a public health issue?**

- Water consumption patterns in the United States have changed in the last several decades, with many people now choosing to purchase bottled water instead of drinking municipal water.

**Public health significance—Why is this work of significance to public health?**

- Because of the cost and other risks associated with bottled water, it is important to determine how much of water consumption patterns are driven by actual risk versus perceived risk and personal preferences.

**Public health implications—What are the key implications or messages for practitioners, policy makers and/or researchers in public health?**

- A greater understanding of decision-making regarding drinking water sources is needed to better support individuals and families in their choice of drinking water.

**Abstract:**

Municipally provided water is low-cost, considered safe in most communities, and usually fluoridated to improve oral health. Yet, many Chicago region families report relying on other water sources. We investigated if safety and quality concerns were associated with these decisions; we also investigated whether there were spatial trends related to lead risk associated with water choice preferences. We used self-reported water consumption behavior data from the Coordinated Oral Health Promotion (CO-OP) Study, a longitudinal cohort of young children and their families. Respondents’ residences (N = 331) were geolocated at the census tract level. We evaluated associations between parent demographics, estimated lead risk and water preferences. Among those who “Never” gave their children tap water, we investigated demographic characteristics associated with viewing tap water as “Not safe”. Sixty-five percent (n = 216) of caregivers report that their child “Never” drinks tap water. Ordinal logistic regression indicates that parents aged <30 years are more likely to respond “Never” relative to “Sometimes” or “Always” (OR = 1.89; CI = 1.04, 3.40). Among those in the “Never” category, we grouped reasons into safety concerns (n = 114), observed quality concerns (n = 48), and preference (n = 40). We found that the decision not to give children municipal water is not aligned with the estimated lead risk. Understanding water consumption choice mechanisms is important for communities seeking safe and quality drinking water.

## 1. Introduction

Trust in municipal tap water has fallen in recent decades [1,2], potentially due in part to awareness of the well-publicized failings of city water systems in some municipalities [3]. This trust issue regarding water safety may be particularly acute for parents because children are uniquely vulnerable to lead [3], an EPA-regulated neurotoxicant that is strongly associated with learning, behavior, and growth problems [3,4,5,6]. No blood lead level has been identified as safe, particularly for young children. The EPA action level for lead in drinking water is 15 µg/L [7]. In Chicago, a city with the greatest number of lead service lines in the United States [8], the city provides support services for those who have documented high lead levels in their homes [9]. The city’s drinking water is regulated by the Department of Water Management [10], which produces reports regarding the quality of the city’s water, noting concentrations of analytes and making these reports available to the public as required by law. Reported in aggregate, the highest lead concentration detected in Chicago’s drinking water was 7.1 ppb (range of detection: 0.69–12 ppb) in 2024 [10], which is below the EPA action level [7]. However, the relationships between lead exposure sources and blood lead level are complex, and there is no scientifically determined health-based action level for water [11]. Since 1996, the imputed prevalence of venous blood lead levels ≥ 5 µg/dL among young children has fallen from 70.2% to 2% in 2019 [12]. To further protect and empower residents, Chicago provides free water lead testing kits and in-home testing to community members [13]. Lead is among the many contaminants that are monitored by the Department of Water Management. These include corrosion (i.e., copper) and disinfectant (i.e., chlorine) contaminants, which can affect water’s appearance and smell/taste [14] but have not been reported at concentrations that violate EPA standards [10]. These data are publicly available and have been used in scholarly research [15].

Despite reassurances of safety and the transparency these data are intended to convey, many in Chicago chose other sources of drinking water, specifically bottled water [15]. The choice of bottled water over municipally provided tap water has been studied widely [16,17,18,19]. The reasons for rejecting tap water in favor of bottled water often highlighted the aesthetic concerns (i.e., smell, taste) regarding tap water, as well as its safety [16,17,20,21]. While many studies of water choice and contaminant exposure risk have been conducted [22,23,24,25,26], they do not focus on young children and their parents who make decisions for them. Further, many of the studies on lead risk in water use secondary data [4], limiting researchers’ ability to ask follow-up questions. Some studies of lead risk are ecological [6,15], preventing causal investigation. To more deeply understand the rationale for children not consuming tap water, we evaluated the water consumption preference data of the COrdinated Oral Promotion (CO-OP) Chicago Study. Grounded in the socioecological framework [27], this longitudinal cohort follows young children over time to address multi-level factors influencing oral health. CO-OP Chicago prioritized recruitment of children living in economically and racially marginalized neighborhoods where dental caries prevalence was highest. Access to low-cost, safe, fluoridated drinking water was one of the variables assessed as part of CO-OP Chicago and forms the basis for this analysis.

Our analysis first tested the hypothesis that parental water choice preferences for their children were associated with parental demographics [17]. For those parents who did not give their children municipal tap water at home, we hypothesized that they had concerns about the safety and aesthetic qualities of water. Further, we hypothesized that safety concerns would be associated with the estimated residential lead risk of the census tracts in which they lived, given that the CO-OP Chicago participant sample lived in neighborhoods with older housing and higher historical lead levels.

## 2. Materials and Methods

### 2.1. Sample

Data from 331 parent–child participant dyads were included (U01DE030067). Focused on economically and racially marginalized communities on the region’s west and south sides, some of the parent–child dyads were recruited from the prior CO-OP Chicago trial [28] (NCT03397589), and others were recruited from the Special Supplemental Nutrition Program for Women, Infants, and Children (WIC) centers and partner community medical clinics. If families did not speak English or Spanish or if the child was designated as a ward of the state, they were not included in the cohort. From 30 March 2022 to 16 May 2023, 331 families were enrolled. Data for this analysis is from the enrollment data collection. Participant data were collected in their homes. The Institutional Review Boards at the University of Illinois Chicago, University of California San Francisco, and the Chicago Department of Public Health approved this study.

### 2.2. Geocoding

Participant baseline addresses were processed through ArcGIS Pro 3.5.2 and ArcGIS StreetMap Premium 2024 Release 2 on TERA, an offline locator, and went through three rounds of geolocation. Addresses were first geocoded as points. Any participants that were not successfully geocoded had their addresses standardized and geocoded using the Street Address category. This limited the locators’ searches, avoided false positive matches, and enabled geocoding at the street address level. Any remaining uncoded addresses were manually geocoded. Collected addresses were entered into the program, and a geolocated point (with a latitude and longitude) was generated using a Geolocation tool. These points were matched to census tracts, which are the level of spatial resolution for this study.

### 2.3. Water Preference Data Collection

Parents were asked, “Do you give faucet water to your children?” Response options were “Always”, “Sometimes”, or “Never”. Only those who reported “Never” drinking water from the tap were asked the reasons why. Response options were as follows: taste, not safe, I was told not to, or other. If caregivers selected “other,” research assistants entered the other reason provided by the parent. Responses were coded by research staff to include new categories based on the “other” responses. A heat map was constructed using Python 7.0.8 to visualize their reason(s) for not drinking tap water and understand points of overlap among them at a sample level. One participant was excluded for not responding to the question.

### 2.4. Dependent Variables

#### Water Preference and Rationale

We evaluated the choice of water for children at the individual level, where it was coded as “Never”, “Sometimes” or “Always.” These choices were coded as “0”, “1” and “2”, respectively; for regression analyses, these choices were reverse-coded to “2”, “1” and “0”, respectively. Separately, we restricted the regression analysis data to those who responded that they “Never” give children water from the tap. The reasons for their choice to give no tap water were grouped thematically for analysis. These domains included the following: safety concerns (“not safe to drink”, “was told not to”, “does not trust the water”, “Chlorine concern”, “Fluoride concern”, “Lead concern”), observation-based concerns (“taste”, “not clean/clear”), and preference (“general preference”, “prefer bottled water”). Participants were able to identify multiple reasons for never giving their children tap water. These responses were coded as “1” for those who identified one of these concerns within a theme and “0” in the absence of a given response. These responses were summed to the census tract level to evaluate the density of the response for spatial analysis.

### 2.5. Exposure Variables

#### 2.5.1. Participant Demographics

During data collection, the parent–child dyad income was categorized into 14 groups, ranging from <USD 5000 to over USD 150,000. For this analysis, we collapsed these groups into a three-level categorized family income variable: <USD 30,000, USD 30,000–USD 59,999, and ≥USD 60,000. Those who responded that they refused to answer, did not know, or “other” to this question were marked as “missing”. Child sex was a native dichotomous variable (male = 0; female = 1). Parent race/ethnicity was a categorical variable (Black/African American; Hispanic/Latino/Latina; Other (White/Caucasian, Asian, Native American/Alaskan Native, Native Hawaiian or Other Pacific Islander; No Primary Affiliation/“Mixed”, Other). “Refused” and “Don’t know” were marked as missing. Parental education was categorized as follows: less than high school; high school degree, GED or vocational education; college experience (some college or associate’s degree or completed Baccalaureate degree, or Master’s or Doctoral degree). “Other” and “Don’t know” were marked as “missing”.

#### 2.5.2. Residential Lead Risk Index

The residential lead risk index is a census tract measure of lead risk constructed from housing and poverty data and gives a general estimate of lead risk for everyone living in a specific census tract. This index does not include blood level data. Following a methodology developed by the Washington Department of Health [29], tract-level American Community Survey housing age and poverty status data at the tract level were downloaded from Census.gov and used to create continuous scores. Scores were then weighed and summed. This index was evaluated against the Chicago Health Atlas Lead Poisoning data [30], comparing the index estimates to the Chicago Department of Public Health blood lead level at the census tract level. We used a Pearson correlation assessment, which produced an ρ_r_ of 0.64 (*p*-value < 0.00). We used the continuous weighted risk score that we produced following the Washington Department of Health/Vox et al.’s methodology [29].

### 2.6. Analysis

#### 2.6.1. Bivariate Analysis

The types of tests used were determined by the variable type and data distributions using standard statistical approaches. Observations with missing data were not included in analyses. To test if lead risk scores, water choice, and reason responses were associated, we first used ANOVA, Kruskal–Wallis and Wilcoxon Rank-Sum tests to determine whether differences in continuous variables (i.e., age, lead risk score) existed among water choice groups in our analytic sample (n = 276); where continuous distributions were not normally distributed, we used nonparametric tests. We then tested for correlations between demographic categorical variables and categorical water choice responses using chi-squared and Fisher’s exact tests to produce RxC tables. We also used chi-squared and Fisher’s exact tests to evaluate correlations between the reasons for not giving children tap water among parents who “Never” gave their children tap water (n = 181). The significance level for all tests is *p* < 0.05.

#### 2.6.2. Regression Analysis

To evaluate the associations between demographics and categorical water choice, we used multivariate ordinal regression [31,32,33], having confirmed the parallel regression assumption using the Brant test. We adjusted for race, income, and parent age. Among the subset who had responded that they “Never” gave their children water from the tap (a dichotomous variable), we performed a multivariate logistic regression to evaluate if “observed quality concerns” were associated with safety concerns, adjusting for parent age. In both regressions, the significance level for these regressions was *p* < 0.05.

Analyses were conducted using Stata 18.0 (College Station, TX, USA).

## 3. Results

### 3.1. Water Choice

#### 3.1.1. Descriptive Findings

The majority of parents reported that their child was of Hispanic race/ethnicity (53.94%) (Table 1). The frequencies of self-reported income categories (<USD 30,000, USD 30,000–USD 59,999 and ≥USD 60,000) were 128 (38.79%), 106 (32.12%) and 45 (13.64%), respectively. The majority of the parents had at least some college education (n = 154; 46.67%). We found that the average lead risk (SD), as assessed by the lead risk metric, was 0.78 (0.73) for the whole sample. Of the 330 parents who responded to the question, “Do you give your child water straight from the faucet?”, 47 (14.24%) parents always did, 67 (20.30%) sometimes did, and 216 (65.45%) never gave their child water straight from the tap to drink (Table 1).

#### 3.1.2. Bivariate Findings: Full Sample

To understand whether there were correlations between water choice responses and the income category in our analytical sample, we performed chi-squared tests and found that income level correlated with water choice (*p* = 0.04) (Table 2). Parent race/ethnicity also correlated with water choice (*p* = 0.01), with Hispanic parents being more likely to “Never” give their children tap water (55.25%). Parent age, in both continuous and categorized form, correlated with water choice. Parents who were 40 years or older were more likely to “Always” give their children water from the tap (36.11% vs. 15.25% vs. 17.13%; *p* < 0.00). There were no differences in lead risk by reported water choice group (Always µ(SD) = 0.70 (0.74), Sometimes µ(SD) = 0.80 (0.68), Never µ(SD)= 0.85 (0.76); *p* = 0.50).

#### 3.1.3. Ordinal Regression

Multivariate ordinal regression was then used to further test the significant associations between water choice and parental demographics identified in the bivariate testing (Table 3). Black parents’ water choices were not different from those of Hispanic parents. The odds of “Never” giving children water directly from the tap compared to those who “Sometimes” or “Always” did was 75% less if the “Other” race/ethnicity category (relative to Hispanic parent ethnicity) was indicated, with other demographic variables held constant. The odds of “Never” giving children water directly from the tap, compared to those who “Sometimes” or “Always” gave their children water directly from the tap, was 1.89 times greater among those parents were younger than 30 years at the time of the survey (relative to those between the ages of 30 and 39), with other variables held constant. The model explained 3% of the variability in the outcome.

### 3.2. “Never” Gives Child Tap Water Subgroup

#### 3.2.1. Descriptive Findings

**Figure 1:**
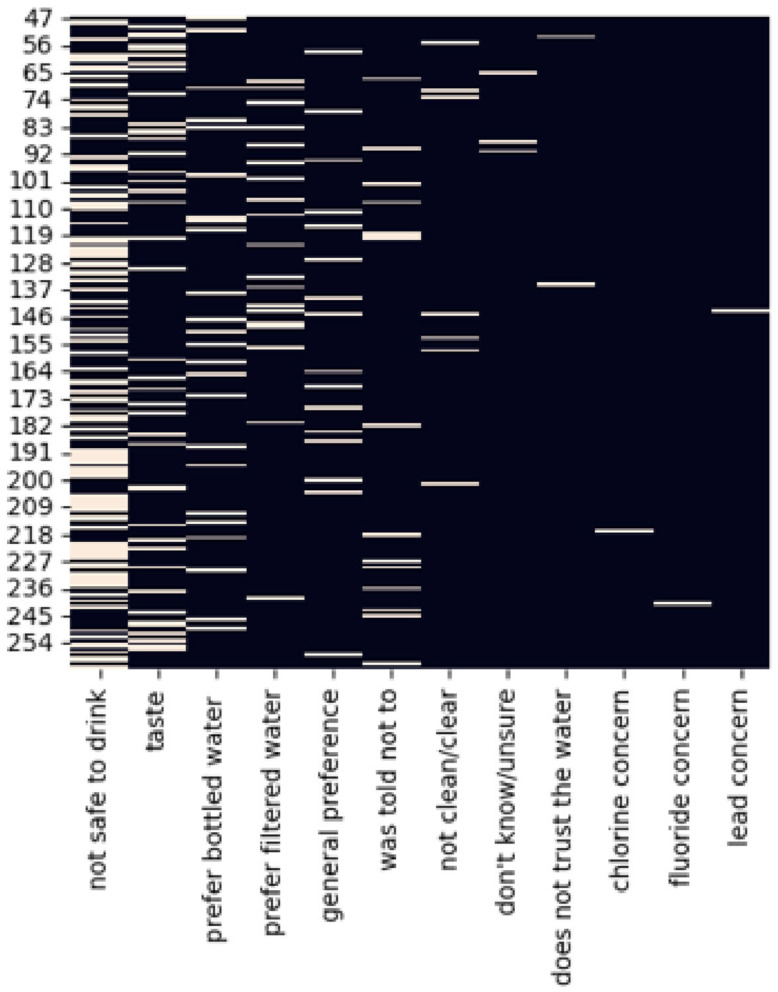
Heat map—reasons for “Never” giving children water directly from the tap. Light bars indicate the participant’s expressed reason for “Never” giving their child tap water.

Of those that did not give their child tap water (n = 216), the frequencies of self-reported income categories (<USD 30,000, USD 30,000–USD 59,999 and ≥USD 60,000) were 92 (42.59%), 65 (30.09%), and 27 (12.50%), respectively (Table 1). The majority of the parents had at least some college education (n = 103; 47.69%). This subsample’s average lead risk (SD), as assessed by the lead risk metric, was 0.79 (0.76). The reasons for not giving tap water were safety concerns (n = 114; 52.78%), observed quality concerns (n = 48; 22.22%), and preference (n = 40; 18.52%). Figure 1 is a heat map that provides a visualization of the full range of reasons reported for never giving tap water and the frequency of the responses. Spatial trends in these responses did not reach statistical significance.

#### 3.2.2. Bivariate Findings

To drill down more about the potential reasons parents “Never” gave their children tap water, we started by testing for a difference within this group by demographic characteristics. We found that, using the Wilcoxon rank-sum test, only the median (range) parent age varied by whether the parent had safety concerns about water (33.49 (22.39, 63.73) vs. 30.8 (22.63, 53.07); *p* = 0.04), with the older group of parents expressing safety concerns (Table 4). We observed no difference between groups for the lead risk index (0.87 (0.78) vs. 0.83 (0.74); *p* = 0.69). Because participants could have multiple reasons for not giving tap water, we tested whether response options were correlated. The majority of participants endorsed just one reason. As would then be expected, those with safety concerns were less likely to also have quality concerns (41.98% vs. 8.0%; *p* = 0.00). Also, caregivers who did not give tap water because of a preference for other sources of water were less likely to also have safety concerns (37.04% vs. 0.00%; *p* = 0.00).

#### 3.2.3. Logistic Regression

Among parents who never gave their children tap water, those who had water quality concerns were much less likely (87% reduced odds) to also have safety concerns (Table 5). Parent age did not significantly associate with safety concerns. The model explained 13% percent of the variation in the outcome.

## 4. Discussion

The results of this study provide insight into some of the potential reasons that many parents (65%) are not giving municipal tap water to their children. Our adjusted model revealed that younger parents (<30 years old) had greater odds of “Never” giving their children tap water. Income was not associated with water choice. Black and Hispanic parents had the same odds of giving their children tap water, but “Other” race/ethnicity parents were less likely to give tap water. When we further investigated trends within the group who “Never” gave their children water straight from the tap, the leading reason in our sample of families of young children in the Chicago region was safety concerns. This concern was not associated with their lead risk as ascertained by a residential lead risk index and was also not associated with their race/ethnicity, income level, or parental educational level. We also found that families generally had just one primary reason (safety, quality, or personal preference) driving their choices.

Previous studies have highlighted the role that water preference plays in people’s consumption of tap water. These studies highlight the organoleptic qualities [14,16,17,20,34] of water that may make the water that comes out of their taps unappealing and may drive parents to drink bottled water and give bottled water to their children. These characteristics fall under the Environmental Protection Agency’s secondary water standards [35,36], which address the technical (produces scaling), aesthetic (aberrant taste, smell), and cosmetic (produces discoloration) qualities of drinking water. These guidelines are unenforceable, however, as they do not pose a health threat to consumers. Our study contributes to the body of literature in that ours focuses on the choices parents make on behalf of their young children, a group whose water consumption is not well studied. We, like the aforementioned researchers, found that qualities observed or preconceived beliefs about tap water may provide sufficient reason not to consume tap water. Community feedback on their experience of drinking water may offer an opportunity for water providers to investigate how to improve the experience of users and increase usage of municipal water. Because of its regulation, municipal water may pose fewer health risks, microplastic exposure, and financial burden than tap water, but this requires additional investigation [37].

### 4.1. Limitations

There are a few notable limitations to our study, primarily that behaviors are self-reported and responses regarding the rationale behind water choices were limited. Participants were not asked follow-up questions about the reasons behind their water choices if they did not respond that they “Never” gave their children tap water. We do not know whether those who “Sometimes” or “Always” gave their children water from the tap did so because they had concerns about safety or not.

Water choices in our sample were not associated with parental income and were slightly associated with race. This result could be due to the homogeneity of our sample; most of the sample was low-income and Black or Hispanic. In other studies with more diversity, Hispanic and African American adults were less likely to drink tap water [19,37]. These studies did not examine parental choice for children. We cannot know if our results represent choices that are unique to Black and Hispanic caregivers in the Chicago region or if they represent larger trends in water choices regarding children. Other studies have found, however, that non-Hispanic Black and Hispanic adults view bottled water as safer and better tasting [16,21].

Among those who responded that they had “Never” given their children tap water, caregivers were not asked what their specific safety concerns were, what quality issues they encountered, or details about their preferences. Even though the question format and delivery allowed parents to endorse multiple concerns, very few did, making it possible that respondents did not fully understand the multiple-choice response option. We also do not know their actual lead or other water risks since their home water was not tested as part of this study. Additionally, our sample was limited to one municipal region and to economically marginalized families.

Though we have rich data at the individual level, we are not able to perform spatial analyses at the individual point level. This imposes some limitations in terms of the analyses we may conduct because individual factors (i.e., reported family income) may not be evaluated spatially as the lead risk index may be. Though we have a robust representation of the sample across our catchment area, some of these tracts are disconnected from each other, which leads some distal areas in Chicago’s collar communities to be excluded from the analysis. This may have impacted our ability to identify spatial trends.

### 4.2. Strengths

Among the strengths of this paper, we had individual-level water preference data. These data provide valuable insights into the reasons why Chicago-area parents are not giving tap water to their children. Further, because these questions about tap water’s safety and quality (appearance) were both asked directly, we minimized the chance that water safety included those responses regarding water’s quality. We were able to investigate whether there were spatial trends present in parents’ water choices and the rationales behind them. Future analyses could integrate publicly available data into our dataset to understand the environmental context that may (or may not) inform parent responses. We found no statistically significant association between the census-tract-level lead risk metric and parents’ water preference for their children—this finding highlights an opportunity for community education about municipal water, what risks may exist, and how to safely manage them.

## 5. Conclusions

Water consumption is increasing, edging out sugary drinks as the beverage of choice in the United States [37]; this is a good thing for the health of all. With this change, the economic and safety factors associated with different sources of water need attention. In our sample of urban economically marginalized families, parents’ decisions to offer their children municipal water were associated with some demographic characteristics. We do not know if parents knew their lead risk, but our results show that census-based lead risk estimates were not associated with parent water choices. Parents who never gave their children tap water were likely to only report one reason, either because of quality or safety concerns. This finding suggests that families fall into distinct groups regarding their beliefs and understandings of the risks posed by different water choices. Further research is needed to understand risk perception and the decision-making process behind the choice to use municipal drinking water. Mixed methods research that involves qualitative components and water testing is important to better understand the decision-making processes parents face regarding water safety. Additionally, greater dialogue among community members, municipal water management officials, social service and housing agencies, and public health officials is important to better understand community water quality concerns and to build trust.

## Figures and Tables

**Table 1:** Descriptive statistics (N = 330).

	Do You Give Your Child Water Directly from the Faucet?			
	Always n = 47 (14.24%)	Sometimes n = 67 (20.30%)	Never n = 216 (65.45%)	Total N = 330 (100%)
Lead Risk Index, Mean (SD)	0.72 (0.66)	0.80 (0.67)	0.79 (0.76)	0.78 (0.73)
Household income				
<USD 5000–≤USD 29,999	13 (27.66%)	23 (34.33%)	92 (42.59%)	128 (38.79%)
≥USD 30,000–≤USD 59,999	12 (25.53%)	29 (43.28%)	65 (30.09%)	106 (32.12%)
≥USD 60,000	11 (23.40%)	7 (10.45%)	27 (12.50%)	45 (13.64%)
Missing	11 (23.40%)	8 (11.94%)	32 (14.81%)	51 (15.45%)
Parent Race/Ethnicity *				
African American/Black	13 (27.66%)	37 (52.22%)	88 (40.74%)	138 (41.82%)
Hispanic/Latino/ Latina	28 (59.57%)	29 (43.28%)	121 (56.02%)	178 (53.94%)
Other *	6 (12.77%)	1 (1.49%)	6 (2.78%)	13 (3.94%)
Missing	0 (0%)	0 (0%)	1 (0.46%)	1 (0.30%)
Parent Education Level				
<High School	4 (8.51%)	6 (8.96%)	31 (14.35%)	41 (12.42%)
High School Degree/GED/Voc. Degree	22 (46.81%)	31 (46.27%)	80 (37.04%)	133 (40.3%)
Some/Completed College Degree/Graduate Degree	21 (44.68%)	30 (44.78%)	103 (47.69%)	154 (46.67%)
Missing	0 (0%)	0 (0%)	2 (0.93%)	2 (0.61%)
Child Sex				
Male	27 (57.45%)	31 (46.27%)	99 (45.83%)	157 (47.58%)
Female	20 (42.55%)	36 (53.73%)	117 (54.17%)	173 (52.42%)
Child Age				
<4 years	3 (6.38%)	7 (10.45%)	31 (14.35%)	41 (12.42%)
4 or more years	44 (93.62%)	60 (89.55%)	185 (85.65%)	289 (87.58%)
Parent Age in Years (Mean, SD)	36.96 (6.88)	32.78 (5.96)	33.41 (6.77)	33.79 (6.74)
Parent Age in Years, Median, (Range)	35.94 (23.37, 55.31)	31.79 (23.1, 46.58)	32.28 (22.39, 63.73)	32.74 (22.39, 63.73)
Parent age				
20–29 years	6 (12.77%)	25 (37.31%)	77 (35.65%)	108 (32.73%)
30–39 years	25 (53.19%)	33 (49.25%)	106 (49.07%)	164 (49.70%)
≥40 years	16 (34.04%)	9 (13.43%)	33 (15.28%)	58 (17.58%)
	Reasons for Not Giving Your Child Water Directly from the Faucet (n = 216)			
Not Safe	114 (52.78%)			
Observed Quality Issues	48 (22.22%)			
Preference	40 (18.52%)			

* White/Caucasian/American Indian/Alaskan Native/Native Hawaiian or Other Pacific Islander/No Primary Affiliation/Mixed/Other.

**Table 2:** Bivariate testing statistics (n = 276).

	Do You Give Your Child Water Directly from the Faucet?			
	Always n = 36 (13.04%)	Sometimes n = 59 (21.38%)	Never n = 181 (65.58%)	*p* Value
Lead risk index, mean (SD)	0.70 (0.74)	0.80 (0.68)	0.85 (0.76)	0.50
Household income				0.04
<USD 5,000–≤USD 29,999	13 (36.11%)	23 (38.98%)	90 (49.72%)	
≥USD 30,000–≤USD 59,999	12 (33.33%)	29 (49.15%)	64 (35.36%)	
≥USD 60,000	11 (30.56%)	7 (11.86%)	27 (14.92%)	
Parent race/ethnicity *				0.01
African American/Black	13 (36.11%)	33 (55,93%)	76 (41.99%)	
Hispanic/Latino/ Latina	18 (50.0%)	26 (44.07%)	100 (55.25%)	
Other *	5 (13.89%)	0 (0.00%)	5 (2.76%)	
Parent education level				0.64
<High School	4 (11.11%)	4 (6.78%)	22 (12.15%)	
High School Degree/GED/Voc. Degree	14 (38.89%)	29 (49.15%)	70 (38.67%)	
Some/Completed College Degree/Graduate Degree	18 (50%)	26 (44.07%)	89 (49.17%)	
Child sex				0.22
Male	22 (61.11%)	26 (44.07%)	84 (46.11%)	
Female	14 (38.89%)	33 (55.93 %)	97 (53.59%)	
Parent age in years, median (range)	36.51 (23.37, 55.31)	33.07 (23.1, 46.58)	32.41 (22.39, 63.73)	0
Parent age				0
20–29 years	2 (5.56%)	20 (33.90%)	65 (35.91%)	
30–39 years	21 (58.33%)	30 (50.85%)	85 (46.96%)	
≥40 years	13 (36.11%)	9 (15.25%)	31 (17.13%)	
Child age				0.26
<4 years	1 (2.78%)	7 (11.86)	22 (12.15%)	
4 or more years	35 (97.22%)	52 (88.14%)	159 (87.85%)	

* White/Caucasian/American Indian/Alaskan Native/Native Hawaiian or Other Pacific Islander/No Primary Affiliation/Mixed/Other.

**Table 3:** Ordinal logistic regression of never choosing tap water (compared to sometimes/always choosing tap water) (n = 276, r^2^ = 0.03).

	Odds Ratio	*p* Value	Lower 95% CI	Upper 95% CI
Household income				
<USD 5000–≤USD 29,999	Ref.			
≥USD 30,000–≤USD 59,999	0.66	0.14	0.38	1.15
≥USD 60,000	0.54	0.09	0.26	1.10
Parent race/ethnicity *				
African American/Black	0.77	0.31	0.46	1.28
Hispanic/Latino/ Latina	Ref.			
Other *	0.25	0.04	0.07	0.96
Parent age				
20–29 years	1.89	0.04	1.04	3.40
30–39 years	Ref.			
≥40 years	0.77	0.42	0.40	1.47

* White/Caucasian/American Indian/Alaskan Native/Native Hawaiian or Other Pacific Islander/No Primary Affiliation/Mixed/Other.

**Table 4:** Bivariate testing statistics for parents who never gave children tap water (n = 181).

	Reported Water Not Safe		
	No n = 81 (44.75%)	Yes n = 100 (55.25%)	*p* Value
Lead risk index, mean (SD)	0.83 (0.74)	0.87 (0.78)	0.69
Household income			0.63
<USD 5000–≤USD 29,999	37 (45.68%)	53 (53.0%)	
≥USD 30,000–≤USD 59,999	31 (38.27%)	33 (33.0%)	
≥USD 60,000	13 (16.05%)	14 (14.0%)	
Parent race/ethnicity *			0.60
African American/Black	35 (43.21%)	41 (41.0%)	
Hispanic/Latino/Latina	45 (55.56%)	55 (55.0%)	
Other *	1 (1.23%)	4 (4.0%)	
Parent education level			0.46
<High School	12 (14.81%)	10 (10.0%)	
High School Degree/GED/Voc. Degree	28 (34.57%)	42 (42.0%)	
Some/Completed College Degree/Graduate Degree	41 (50.62%)	48 (48.0%)	
Child sex			0.06
Male	44 (54.52%)	40 (40.0%)	
Female	37 (45.68%)	60 (60.0%)	
Parent age in years, median (range)	30.8 (22.63, 53.07)	33.49 (22.39, 63.73)	0.04
Parent age			0.16
20–29 years	35 (43.21%)	30 (30.0)	
30–39 years	35 (43.21%)	50 (50.0%)	
≥40 years	11 (13.58%)	20 (20.0%)	
Child age			0.15
<4 years	13 (16.05%)	9 (9.0%)	
4 or more years	68 (83.95%	91 (91.0%)	
Observed quality concerns			0
No	47 (58.02%)	92 (92%)	
Yes	34 (41.98%)	8 (8.0%)	
Preference			0
No	51 (62.96%)	100 (100.0%)	
Yes	30 (37.04%)	0 (0.0%)	

* White/Caucasian/American Indian/Alaskan Native/Native Hawaiian or Other Pacific Islander/No Primary Affiliation/Mixed/Other.

**Table 5:** Logistic regression testing for associations between parent-reported reasons for never giving children tap water (n = 181, r^2^ = 0.13).

Water Not Safe	Odds Ratio	*p* Value	Lower 95% CI	Upper 95% CI
Observed Quality Concerns	0.13	0.00	0.06	0.31
Parent Age (Continuous)	1.03	0.28	0.98	1.08

## Data Availability

When the primary analyses and funding period are complete, data will be made publicly available. At this time, data are available upon request to CO-OP’s principal investigator.

## References

[1] Swanson E. (2016). AP-GfK Poll: About Half of Americans Confident in Tap Water. AP News. https://apnews.com/general-news-eedf886daa334d7c871e531d804620a1.

[2] Water Quality Association (2023). Summary & Highlights: National Study of Consumers’ Opinions & Perceptions Regarding Water Quality, 2023 Edition. https://wqa.org/wp-content/uploads/2023/06/Consumer-Opinion-Study-2023-Public.pdf.

[3] Ortega D.R., Esquivel D.F.G., Ayala T.B., Pineda B., Manzo S.G., Quino J.M., Mora P.C., de la Cruz V.P. (2021). Cognitive Impairment Induced by Lead Exposure during Lifespan: Mechanisms of Lead Neurotoxicity. Toxics.

[4] Callender R., Tootoo J., Miranda M.L. (2024). Using Geospatial Methods in Childhood Lead Poisoning Prevention Programs. Rev. Environ. Econ. Policy.

[5] Miranda M.L., Dolinoy D.C., Overstreet M.A. (2002). Mapping for prevention: GIS models for directing childhood lead poisoning prevention programs. Environ. Health Perspect..

[6] Stanek L.W., Xue J., Zartarian V.G., Poulakos A.G., Tornero-Velez R., Snyder E.G., Walts A., Triantafillou K. (2024). Identification of high lead exposure locations in Ohio at the census tract scale using a generalizable geospatial hotspot approach. J. Expo. Sci. Environ. Epidemiol..

[7] Agency for Toxic Substance and Disease Registry (2023). Lead (Pb) Toxicity: What Are U.S. Standards for Lead Levels?. https://archive.cdc.gov/www_atsdr_cdc_gov/csem/leadtoxicity/safety_standards.html.

[8] Alkafaji R. (2023). Top 10 Cities with the Most Lead Pipes. https://blogs.edf.org/health/2023/06/06/top-10-cities-with-lead-pipes/.

[9] City of Chicago Health Alert Network (2025). Lead Poisoning Prevention—HAN—Chicago Health Alert Network. HAN. https://www.chicagohan.org/programs/lead.

[10] City of Chicago (2025). Water Quality Results and Reports. https://www.chicago.gov/content/city/en/depts/water/supp_info/water_quality_resultsandreports.html.

[11] Chicago Department of Public Health (2023). Childhood Lead Poisoning Prevention & Data Report, 2019–2021. https://www.chicago.gov/content/dam/city/depts/cdph/health_data/CDPH-020_LeadReport_DataReport_R4b_digital.pdf.

[12] CDC (2024). CDC Updates Blood Lead Reference Value. Childhood Lead Poisoning Prevention. https://www.cdc.gov/lead-prevention/php/news-features/updates-blood-lead-reference-value.html.

[13] (2023). Chicago City Hall. Water Lead Test Kit. https://311.chicago.gov/s/article/Water-Lead-Test-Kit?language=en_US.

[14] Dietrich A.M., Burlingame G.A. (2015). Critical Review and Rethinking of USEPA Secondary Standards for Maintaining Organoleptic Quality of Drinking Water. Environ. Sci. Technol..

[15] Huynh B.Q., Chin E.T., Kiang M.V. (2024). Estimated Childhood Lead Exposure From Drinking Water in Chicago. JAMA Pediatr..

[16] Colburn A.T., Kavouras S.A. (2021). Tap Water Consumption and Perceptions in United States Latinx Adults. Nutrients.

[17] Delpla I., Legay C., Proulx F., Rodriguez M.J. (2020). Perception of tap water quality: Assessment of the factors modifying the links between satisfaction and water consumption behavior. Sci. Total Environ..

[18] Bear S.E., Waxenberg T., Schroeder C.R., Goddard J.J. (2024). Bottled water, tap water and household-treated tap water–insight into potential health risks and aesthetic concerns in drinking water. PLoS Water.

[19] Rosinger A.Y., Patel A.I., Weaks F. (2022). Examining recent trends in the racial disparity gap in tap water consumption: NHANES 2011–2018. Public Health Nutr..

[20] Doria M.d.F., Pidgeon N., Hunter P.R. (2009). Perceptions of drinking water quality and risk and its effect on behaviour: A cross-national study. Sci. Total Environ..

[21] Park S., Onufrak S.J., Cradock A.L., Patel A., Hecht C., Blanck H.M. (2023). Perceptions of Water Safety and Tap Water Taste and Their Associations With Beverage Intake Among U.S. Adults. Am. J. Health Promot..

[22] Graydon R.C., Gonzalez P.A., Laureano-Rosario A.E., Pradieu G.R. (2019). Bottled Water versus Tap Water: Risk Perceptions and Drinking Water Choices at the University of South Florida. Int. J. Sustain. High Educ..

[23] Javidi A., Pierce G.U.S. (2018). Households’ Perception of Drinking Water as Unsafe and its Consequences: Examining Alternative Choices to the Tap. Water Resour. Res..

[24] Qian N. (2018). Bottled Water or Tap Water? A Comparative Study of Drinking Water Choices on University Campuses. Water.

[25] Grupper M.A., Schreiber M.E., Sorice M.G. (2021). How Perceptions of Trust, Risk, Tap Water Quality, and Salience Characterize Drinking Water Choices. Hydrology.

[26] Olagunju K., Sante M.R., Bracey G., Greenfield B.K. (2023). Bottled vs tap water perceptions, choices and recommendations in a US Midwest university community. Int. J. Sustain. High Educ..

[27] Fisher-Owens S.A., Gansky S.A., Platt L.J., Weintraub J.A., Soobader M.-J., Bramlett M.D., Newacheck P.W. (2007). Influences on children’s oral health: A conceptual model. Pediatrics.

[28] Martin M.A., Zimmerman L.J., Rosales G.F., Lee H.H., Songthangtham N., Pugach O., Sandoval A.S., Avenetti D., Alvarez G., Gansky S.A. (2020). Design and sample characteristics of COordinated Oral health Promotion (CO-OP) Chicago: A cluster-randomized controlled trial. Contemp. Clin. Trials.

[29] Washington State Department of Health A Targeted Approach to Blood Lead Screening in Children, Washington State: 2015 Expert Panel Recommendations. 2015 Nov. Report No.: DOH 334-383. https://s3.documentcloud.org/documents/2644455/Expert-Panel-Childhood-Lead-Screening-Guidelines.pdf.

[30] Lead Poisoning Rate. Chicago Health Atlas. https://chicagohealthatlas.org/indicators/LDPP.

[31] Williams R. (2005). Models for Ordinal Dependent Variables. https://academicweb.nd.edu/~rwilliam/gologit2/NASUG2005.pdf.

[32] Tutz G. (2022). Ordinal regression: A review and a taxonomy of models. WIREs Comput. Stat..

[33] Agresti A., Tarantola C. (2018). Simple ways to interpret effects in modeling ordinal categorical data. Stat. Neerl..

[34] Domoń A., Kowalska B., Papciak D., Wojtaś E., Kamińska I. (2024). Safety of Tap Water in Terms of Changes in Physical, Chemical, and Biological Stability. Water.

[35] CDC (2024). Drinking Water Standards and Regulations: An Overview. Drinking Water. https://www.cdc.gov/drinking-water/about/drinking-water-standards-and-regulations-an-overview.html.

[36] IL EPA (2025). Drinking Water Branch. https://water.epa.state.il.us/dww/index.jsp.

[37] Vieux F., Maillot M., Rehm C.D., Barrios P., Drewnowski A. (2020). Trends in tap and bottled water consumption among children and adults in the United States: Analyses of NHANES 2011–16 data. Nutr. J..

